# Circumscribed Pneumonia of the Left Lung, Terminating in Gangrenous Abscess; Recovery; Signs of Cavity, with Double Musical Murmur, Excited by the Heart’s Action; Latent Tubercles at the Apex of the Right Lung

**Published:** 1846-05

**Authors:** C. M. Durrant


*Circumscribed Pneumonia of the Left Lung, Terminating in Gangrenous Abscess; Recovery; Signs of Cavity, with double Musical Murmur, Excited by the Heart's Action; Latent Tubercles at the apex of the right Lung.* By C. M. Durrant, M. D.—A young man, after prolonged exposure to cold in an open boat, returned home chilly and shivering. On the following morning he complained of dull pain situated about two inches above the left mamma, and slightly aggravated by a deep inspiration; some cough, without expectoration; pyrexia; pulse 80; bowels regular. Ten days after this, (the period at which I first saw him in conjunction with his usual attendant,) the countenance was anxious and sallow; tongue clean; pulse 80, of moderate strength; there existed also pain over the left mammary region, with cough and muco-purulent expectoration. Has never had hæmoptysis, but has lost a sister from pulmonary phthisis.

*Physical signs.* Chest expands freely.—*Percussion:* Complete dulness to the extent of from two to three square inches over the painful spot above, and a little to the sternal side of the lift mamma; a flat rather than a dull sound was also elicited under the right clavicle. —*Auscultation:* Total absence of breath-sound over the consolidated spot, with a small crepitating rhonchus around its edges; increased resonance of voice and cough, with loud transmission of the sound of the heart through the diseased portion of lung. Respiratory murmur feeble at right apex in front.

Throughout the treatment of this case the system resisted the influence of mercury, the gums remaining unaffected notwithstanding the most diligent and prolonged internal and external exhibition of the remedy. A similar difficulty occurred in exciting external inflammation over the diseased spot by means of counter-irritation.

The patient continued for some weeks much in the same condition, with cough, muco-purulent expectoration, acceleration of pulse, and emaciation. The appetite was good, but the defined dulness, with extinction of breath-sound, remained unaltered. At this period the expectoration assumed a dark greenish yellow colour, both it and the breath of the patient becoming at the same time intolerably fœtid. Percussion now yielded a clearer sound; a large gurgling rhonchus occupying the previously dull spot.

Under these circumstances the prognosis became in the highest degree unfavourable, since, in addition to the abscess, indubitable signs of latent tubercles were evident at the apex of the opposite lung. The appetite, happily continued tolerably good, a free allowance of nutriment was taken. The fœtor of breath was removed by the inhalation of creosote, commencing with one drop three times a day, increased to four, above which quantity it was not well borne. Iron and quinine, with the iodide of potassium and dilute nitric acid, were at the same time prescribed.

Under this treatment, the features of the case assumed a more favourable aspect; the cough and expectoration diminished; the gurgling rhonchus was succeeded by cavernous breathing, and pectoriloquy; the pulse at the same time becoming gradually reduced in frequency. The shower bath was ultimately used daily with decided benefit. Throughout the progress of the case, the patient remained free from perspiration.

Two years have elapsed since the occurrence of the phenomena to which the above report refers; the patient, notwithstanding one or two very slight attacks of hæmoptysis, has continued to feel and believe himself well. An opportunity having presented within the last few weeks, (Nov. 25th, 1845,) of again examining the chest, I am enabled to append the following note of his present physical condition.

States that he feels well, has gained flesh, and is free from cough. Continues to use the shower-bath daily.—*Physical Signs:* Infra clavicular region of the right side expands slightly less than the left. —*Percussion:* Stroke sound under the right clavicle flat, scarcely amounting to dulness; normal on the left side; loud amphoric sound elicited between the third and fourth ribs of the same side; resonance healthy over the remainder of the chest.—*Auscultation:* Respiratory murmur feeble under the right clavicle, confined to the apex of the lung; healthy behind; resonance of voice not increased; cavernous breathing, with pectoriloquy audible over a spot about an inch in diameter, corresponding to the seat of the amphoric stroke-sound, and accompanied by a prolonged double musical murmur, excited by each action of the heart, and strictly confined to the site of the cavity. On removing the stethoscope to the extent of an inch in any direction beyond the walls of the cavity, the sound is rendered at once inaudible.

This case presents many points of interest. The patient, in previous good health, exposes himself upon the water to cold, returns home chilled, has a rigor, followed by general febrile symptoms, with pain and other phenomena sufficiently evident to direct attention to the chest, as the principal seat of the disease. The *pain* was located in one spot, and persistent; it was described as a dull pain, and not increased by motion or intercostal pressure. These characters, while they negatived the existence of pleurodynia, as well as pleurisy, rendered at the same time highly probable the presence of pneumonia. The correctness of this opinion was fully realised by the character of the physical signs, which, throughout the progress of the case, were exceedingly well marked. The dulness on percussion, to the extent of from two to three square inches, situated over the locality of the pain, at once indicated the existence of a circumscribed portion of consolidated lung; and that pneumonia was the disease upon which this sign depended, was further evidenced by the simultaneous occurrence of a fine crepitation around its edges. As the disease, instead of undergoing resolution, advanced to abscess, the physical phenomena became equally indicative of this more serious lesion. We now find, over the previously dull spot, an amphoric sound, elicited by percussion, pointing to the existence of a cavity communicating freely with a bronchial tube; while on auscultation a large gurgling rhonchus, corresponding to the spot over which the respiratory murmur was before inaudible, became at the same time painfully distinct. These circumstances, together with the history of the case, and the characteristic odour and appearance of the expectoration, rendered the nature of the disease unequivocally apparent.

The object of the early treatment, viz., the resolution of the consolidated portion of lung, was frustrated by the resistance offered by the system to the introduction of mercury, as well as the great difficulty which existed in producing counter-irritation over the affected part. It was omitted in the report, but which may now be stated, that a seton introduced in the mammary region, was equally unsuccessful in effecting a discharging surface.

This antagonism between internal disease, and the operation of remedies, is not uncommon, more especially when the former is confined to a small spot, and assumes from the first a sub-acute or chronic character. I have, however, in no other instance witnessed so persistent a determination (so to speak) of the system to resist remedial measures as in the present case.

On the formation of abscess, the most marked benefit was derived from the inhalation of creosote. It removed the fœtor from the expectoration; it lessened the cough, and greatly diminished the amount of expectoration.

On the present condition of the case, it is necessary to make one or two observations.

The patient considers himself strong and well, and notwithstanding the most rigid injunctions on the part of his medical attendant, he unfortunately evinces extreme imprudence in reference to exposure to cold. The amphoric stroke-sound, together with the perfect pectoriloquy that now exists, indicate a small cavity in the original site of the abscess, lined probably by organized membrane. Its proximity to the heart causes, at each pulsation of that organ, a vibration of the air within the cavity, and thus gives rise to the double musical murmur so distinctly heard, yet so completely circumscribed in extent. This latter sound is unquestionably of rare occurrence, requiring for its production a cavity containing air only, of certain dimensions, communicating with the bronchi, and at the same time sufficiently near the heart, as to be influenced by its movements.

The pulsatory gurgling sound pointed out by Dr. Stokes, arising from the agitation of fluid in cavities, excited by the action of the heart, will be met with more frequently than the phenomenon of the double murmur above mentioned. Of the condition of the apex of the right lung no doubt can longer exist in reference to the presence of tubercles. When the patient first came under observation, he had had no hæmoptysis; the result of percussion was a flat, rather than a dull sound; and the only indication derivable from auscultation was a slight feebleness in the respiratory murmur. Under these circumstances, therefore, with a deranged condition of the circulation in the left lung, the physical signs alluded to might possibly have arisen from congestion, and a doubtful opinion upon this point was therefore expressed. Now, however, two years having elapsed, and the same phenomena obtaining, together with the fact, that a small quantity of blood has more than once been observed in the sputa, coupled with the recollection of a sister having died of phthisis, I have no hesitation in pronouncing, that latent tuberculization exists at the apex of that lung. The fact of the deposit, which is scattered, having neither increased nor excited surrounding inflammatory action, is unquestionably a favourable circumstance; still, however, in a case presenting similar conditions, even under the best directed auspices for the future, a rigidly guarded prognosis should be given.—*Ibid.*

